# Current Research in Lidar Technology Used for the Remote Sensing of Atmospheric Aerosols

**DOI:** 10.3390/s17061450

**Published:** 2017-06-20

**Authors:** Adolfo Comerón, Constantino Muñoz-Porcar, Francesc Rocadenbosch, Alejandro Rodríguez-Gómez, Michaël Sicard

**Affiliations:** 1 Remote Sensing Laboratory, Universitat Politècnica de Catalunya, 08034 Barcelona, Spain; constan@tsc.upc.edu (C.M.-P.); roca@tsc.upc.edu (F.R.); alejandro@tsc.upc.edu (A.R.-G.); msicard@tsc.upc.edu (M.S.); 2 Ciències i Tecnologies de l’Espai–Centre de Recerca de l’Aeronàutica i de l’Espai/Institut d’Estudis Espacials de Catalunya (CTE-CRAE/IEEC), Universitat Politècnica de Catalunya, 08034 Barcelona, Spain

**Keywords:** aerosol, lidar, review, technology

## Abstract

Lidars are active optical remote sensing instruments with unique capabilities for atmospheric sounding. A manifold of atmospheric variables can be profiled using different types of lidar: concentration of species, wind speed, temperature, etc. Among them, measurement of the properties of aerosol particles, whose influence in many atmospheric processes is important but is still poorly stated, stands as one of the main fields of application of current lidar systems. This paper presents a review on fundamentals, technology, methodologies and state-of-the art of the lidar systems used to obtain aerosol information. Retrieval of structural (aerosol layers profiling), optical (backscatter and extinction coefficients) and microphysical (size, shape and type) properties requires however different levels of instrumental complexity; this general outlook is structured following a classification that attends these criteria. Thus, elastic systems (detection only of emitted frequencies), Raman systems (detection also of Raman frequency-shifted spectral lines), high spectral resolution lidars, systems with depolarization measurement capabilities and multi-wavelength instruments are described, and the fundamentals in which the retrieval of aerosol parameters is based is in each case detailed.

## 1. Introduction

Light detection and ranging (lidar) sensors are the counterpart of radar sensors working in the optical wavelength spectral range. In this spectral range the interactions between electromagnetic radiation and atmospheric constituents are strong, making it possible to detect the presence and concentration of aerosols and trace gases [1] and measure the speed of wind [2] with high spatial resolution. Over the last four decades the lidar technique has proved to be an efficient tool for evaluating the stratification of aerosols, i.e., the vertical structure of the aerosol layers (base, top and thickness) and in particular of the planetary boundary layer (PBL). Elastic (emission and reception are done at the same wavelength) and Raman (the reception wavelength is shifted relative to that emitted by the Raman effect) lidar systems allow the measurement of optical properties such as the backscatter and extinction coefficients. Advanced lidar systems (at least three elastic channels and two Raman channels) also provide information on the aerosol microphysical properties (effective radius, single scattering albedo, modal volume concentration, complex refractive index). Table 1 summarizes the main properties of atmospheric aerosols that can be retrieved with aerosol lidar systems and the minimum configuration needed for it. Based on Table 1, one sees that the retrieval of structural and optical properties, which requires simple systems (one or two channels), dates back to the sixties and seventies of the 20th century, while the retrieval of microphysical properties, which requires advanced systems, is much more recent (~year 2000). In this field scientific breakthroughs are strongly related to the technological progress, and especially to the emission (laser) and reception sub-systems, which are the two backbones of a lidar instrument.

Although progress in laser and detector technologies has been continuous over the last four decades, present-day lidar sensors can still be improved to continue contributing to answering the major scientific questions of atmospheric sciences. In particular, the contribution of atmospheric aerosols is still poorly quantified in some areas of atmospheric sciences because of the large uncertainties associated to the retrieval of some of their properties. As an example, the Intergovernmental Panel on Climate Change (IPCC) has emphasized in each of its reports since 2001 [27] our lack of knowledge of the aerosol impact on the Earth radiation budget and on the climate. The assessment of the effects of aerosols on climate must take into account the large spatial and temporal variations of their properties and of their concentration in the atmosphere. Determining the average global aerosol radiative forcing is necessary but not sufficient because of these large variations (which is not the case for greenhouse gases such as carbon dioxide and methane, which are more uniformly distributed). The difficulties in quantifying the contribution of aerosols, not only locally but also globally, are based on:

- their high variability in space and time and, as a consequence, on their non-localized distribution, mostly due to medium- and long-range transport and short mean life time;
- the geographical extension of the sources: some are localized, others are distributed over large volumes;
- the large number of processes that lead to their production;
- the numerous and heterogeneous processes through which aerosols can interact during their lifetime: nucleation, condensation, coagulation and deposition.

These difficulties have led the aerosol community to start deploying lidar systems not only on the ground but also in space to get a better global coverage of the vertical distribution of aerosol properties. The Lidar In-Space Technology Experiment (LITE) instrument, a 3β system launched in 1994 by the U.S. National Aeronautics and Space Administration (NASA) onboard the Discovery space shuttle, was the first one of the kind. Currently two lidars are operating in space: Cloud-Aerosol Lidar with Orthogonal Polarization (CALIOP), a 2β + 1δ system onboard the Cloud-Aerosol Lidar and Infrared Pathfinder Satellite Observations (CALIPSO) satellite, and Cloud-Aerosol Transport System (CATS), a system onboard the International Space Station (ISS) able to operate in different modes (2β + 2δ, 2β + 1α + 2δ). Future spaceborne lidar missions are: Atmospheric Dynamics Mission-Aeolus (ADM-Aeolus) from the European Sapce Agency (ESA), Earth Cloud Aerosol and Radiation Explorer (EarthCARE) from ESA and the Japan Aerospace Exploration Agency (JAXA), and Advanced Composition Explorer (ACE) from NASA.

This review aims at providing the state of the art of the lidar technology used for the remote sensing of atmospheric aerosols. A short section first presents general concepts about lidar sensors and the aerosol optical properties involved in lidar measurements. Then the technological review is presented from simple to advanced systems and structured according to the following sub-sections: 1β elastic system (including ceilometers), 1β + 1α vibro-rotational and pure rotational Raman system, high spectral resolution lidar (HSRL) system, depolarization-sensitive system and multi-wavelength system. In each sub-section, after the description of the technology, a brief discussion explains how the aerosol properties are retrieved from the lidar signal.

## 2. General Concepts about Lidar Sensors and Aerosol Optical Properties Involved in Lidar Measurements

**Figure 1:**
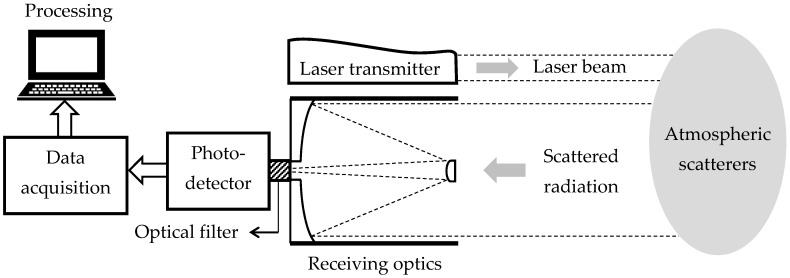
Basic schematics of a lidar system: A laser transmitter emits light pulses to the atmosphere; an optical assembly, usually a telescope, collects part of the scattered radiation, which, after being filtered, is brought onto a photo-detector; the detected signal is then amplified, digitized and processed to retrieve atmospheric parameters.

Lidar systems consist in general of co-located transmitter and receiver sub-systems, with fast photodetectors and acquisition electronics. The basic layout of a lidar is shown in Figure 1. For aerosol remote sensing the transmitter is usually a pulsed laser and the receiver an optical assembly with a telescope which acts as a collector of the backscattered radiation. The optical signal is then filtered to reduce the background-radiation induced noise, converted into an electric signal by means of a fast photodetector, amplified, digitized and recorded for later processing.

The recent progress in aerosol lidars is greatly due, on the side of the laser source, to the availability of efficient and reliable lasers (mainly Nd: YAG lasers, with fundamental wavelength at 1064 nm), whose infrared output radiation frequency can be efficiently doubled and tripled using non-linear crystals, resulting in effective three-frequency sources with wavelengths conveniently located in the near infrared, the visible and the near ultraviolet. On the receiver side, the combination of sensitive, compact and reliable photodetectors (avalanche photodiodes and photomultiplier tubes), of narrowband, custom-made interference filters, and of fast and flexible acquisition systems allows for sensitive—in some conditions only limited by the signal own shot noise—detection, and storage of the faint returns from the atmosphere.

The atmospheric constituents (aerosols and molecules) play two roles: they attenuate the emitted radiation along its path in the atmosphere and they cause a part of the incident radiation to be scattered back towards the receiving optics.

The transmittance $T_{\lambda_{0}}\left(R\right)$ of an atmospheric path of length *R* can be written [28] as:

(1) $$T_{\lambda_{0}}\left(R\right)=\frac{I_{\lambda_{0}}\left(R\right)}{I_{\lambda_{0}}\left(0\right)}=\exp\left[-\int_{0}^{R}\alpha_{\lambda_{0}}\left(u\right)du\right]$$

where $I_{\lambda_{0}}\left(R\right)$ is the intensity (W) at range *R* (m), $\lambda_{0}$ is the operating wavelength (m), and $\alpha_{\lambda_{0}}$ is the total atmospheric optical extinction coefficient (m^−1^). Because of the different extinction mechanisms, the extinction coefficient is a composite phenomenon merging into a single body scattering and absorption coefficients. Extinction is produced by aerosols and molecules. Hence, the total extinction coefficient can be expressed as:

(2) $$\alpha_{\lambda_{0}}=\alpha_{\lambda_{0}}^{aer}+\alpha_{\lambda_{0}}^{mol}$$

where the superscripts “*aer*” and “*mol*” stand for “aerosols” and “molecules”, respectively.

The backscattering phenomenon is parametrized by the atmospheric backscatter coefficient, $\beta_{\lambda_{0}}$ (m^−1^·sr^−1^), which is also caused by aerosols and molecules ($\beta_{\lambda_{0}}=\beta_{\lambda_{0}}^{aer}+\beta_{\lambda_{0}}^{mol})$. The backscatter coefficient quantifies the radiation scattered in the direction reverse to that of the incident radiation.

Under multiple scattering conditions, which are usually associated to optically dense atmospheres and wide field of views, all lidar equations of Section 3 can be adapted by including a correction factor [29].

## 3. Typical Configurations of Aerosol Lidars

### 3.1. 1β Elastic (Including Ceilometers)

1β elastic lidar refers to systems emitting at a single wavelength and designed to detect only the elastic return, i.e., the one in which the energy of the incident photons is conserved. The elastic-backscatter single-scattering lidar equation takes the form:

(3) $$P_{\lambda_{0}}\left(R\right)=\frac{K_{\lambda_{0}}}{R^{2}}\left[\beta_{\lambda_{0}}^{aer}\left(R\right)+\beta_{\lambda_{0}}^{mol}\left(R\right)\right]T_{\lambda_{0}}^{2}\left(R\right)O_{\lambda_{0}}\left(R\right)$$

where $P_{\lambda_{0}}\left(R\right)$ is the backscattered power (W) received from range *R* and $K_{\lambda_{0}}$ is the lidar system constant ($K_{\lambda_{0}}=E\left(\lambda_{0}\right)A_{r}\xi \left(\lambda_{0}\right)c/2)$ where $E\left(\lambda_{0}\right)$ is the pulse energy (J) at wavelength $\lambda_{0}$, $A_{r}$ the effective telescope receiving area (m^2^), $\xi \left(\lambda_{0}\right)$ the optics net transmission of the system [.], and *c* the speed of light (m·s^−1^). The term $T_{\lambda_{0}}^{2}\left(R\right)$ accounts for the two-way path atmospheric transmittance due to both aerosols and molecules. $O_{\lambda_{0}}\left(R\right)$ is the overlap function, inherent to any lidar, taking into account the unit-normalized cross-over function between the laser-illuminated atmospheric cross-section at a range *R* and the telescope field of view. It depends on many different optical and geometrical parameters of the system as well as on the laser intensity distribution (irradiance) of the beam [30,31,32,33].

The solution of the lidar equation stands for independent retrieval of the profiles of the extinction and backscatter coefficients. In an elastic lidar system, this is an underdetermined problem because it involves retrieval of two unknowns ($\alpha_{\lambda_{0}}\left(R\right)$ and $\beta_{\lambda_{0}}\left(R\right)$) from a single observable, $P_{\lambda_{0}}\left(R\right)$ [34].

Historically, the solution of the elastic lidar equation started as early as 1954 with the efforts of Hitschfeld and Bordan [35] to invert the rain rate from radar returns, which was later revisited to invert the lidar equation by Fernald et al. [6], among others. Kaul in 1976 [36] and later Klett in 1981, presented a stable backward solution [37] of the lidar equation that assumed a *one-component* atmosphere (i.e., no separation between aerosol and molecular components). Central to this step was a reformulation of the lidar equation in differential form by using the elementary structure of Bernoulli’s equation [38]. In 1984, Fernald [7] published the *two-component* version of the elastic-lidar inversion algorithm, which Klett [8] reformulated in a unified approach (Klett-Fernald’s (KF) algorithm in what follows). Irrespective of whether the one- or the two-component solution forms are considered, two inputs are necessary from the user’s side to cope with the inherent under-determination of the lidar equation: (i) a boundary calibration, usually in the form of a far-end backscatter-coefficient calibration; and (ii) an extinction-to-backscatter relation. This is detailed next, in the context of today’s standard form of the two-component elastic-lidar inversion algorithm. The backward form of the KF algorithm for the retrieval of the aerosol backscatter coefficient at $\lambda_{0}$ takes the form [39]:

(4) $$\beta^{aer}\left(R,\beta_{m},{\vec{S}}^{aer},\vec{U}\right)=\frac{U\left(R\right)F\left(R,{\vec{S}}^{aer}\right)}{\frac{U_{m}}{\beta_{m}}+2\int_{R}^{R_{m}}S^{aer}\left(v\right)U\left(v\right)F\left(v,{\vec{S}}^{aer}\right)dv}-\beta^{mol}\left(R\right),\;R\leq R_{m}$$

where $F\left(R,{\vec{S}}^{aer}\right)=\exp\left\{2\overset{R_{m}}{\underset{R}{\int }}\left[S^{aer}\left(u\right)-S^{mol}\right]\beta^{mol}\left(u\right)du\right\},\;U\left(R\right)=U^{2}P\left(R\right)$, is the range-corrected lidar return power, $S^{aer}\left(R\right)=\alpha^{aer}\left(R\right)/\beta^{aer}\left(R\right)$ is the aerosol extinction-to-backscatter ratio (so-called aerosol lidar ratio), $S^{mol}=8\pi /3$ is the molecular lidar ratio, $R_{m}$ is the far-end calibration range, $\beta_{m}$ and $U_{m}$ are shorthand notations for $\beta \left(R_{m}\right)$ and $U\left(R_{m}\right),$ respectively. Vector notation, ${\vec{S}}^{aer}$ and $\vec{U}$, has been used to represent range-dependent functions, $S^{aer}\left(R\right)$ and *U*(*R*), and the subscript $\lambda_{0}$ has been dropped to shorten the notation.

In Equation (4) $\beta_{m}\;\left(\beta_{m}=\beta^{aer}\left(R_{m}\right)+\beta^{mol}\left(R_{m}\right)\approx \beta^{mol}\left(R_{m}\right)\right)$ and $\beta^{mol}\left(R\right)$ are estimated from local radiosonde pressure/temperature measurements or by resorting to a standard atmosphere model [40]. The aerosol lidar ratio, $S^{aer}\left(R\right)$ is usually assumed to be known and range independent by using previous knowledge of the type of aerosol to be measured [41,42,43], which is questionable in the case of complex layering of aerosols [44]. The works [45,46] show the importance of reducing the noise in *U_m_* by spatially averaging around the calibration range to reduce inversion error bars. A unified study on the impact of both random and systematic error sources and their spectral dependency can be found in [39]. The solution given by Equation (4) can also be found in a less formal, yet numerically equivalent, way taking into account that Equation (3) implies that $\beta^{aer}\left(R\right)=\beta_{m}\frac{U\left(R\right)}{U_{m}}exp\left\{-2\int_{R}^{R_{m}}\left[\alpha^{aer}\left(x\right)+\alpha^{mol}\left(x\right)\right]dx\right\}-\beta^{mol}\left(R\right).$ Then $\beta^{aer}\left(R\right)$ can be found iteratively [47], starting with an initial guess of $\alpha^{aer}\left(R\right)$; this results in a first guess for $\beta^{aer}\left(R\right)$, which, using the assumed $S^{aer}\left(R\right)$ yields a refined $\alpha^{aer}\left(R\right)$ serving to calculate another iteration of $\beta^{aer}\left(R\right)$. Iteration cycles are pursued until differences between the optical coefficients in successive iterations are negligible.

Though historically the elastic-lidar inversion algorithm was formulated in extinction terms, trustworthy extinction profiles are difficult to achieve. This is because the extinction profile must be determined by multiplying the retrieved backscatter profile (Equation (4)) with the lidar ratio profile used before as input in the algorithm [34]. Alternative methods allowing extinction retrievals have been proposed by replacing the far-end boundary calibration by the optical thickness measured along the sounding path (e.g., by using sun-photometer AErosol Robotic NETwork—AERONET— measurements) [48,49], by a near-end calibration measured with a nephelometer [50] or by combining multi-angle measurements at a constant azimuth [51]. The latter requires the assumption of a horizontally stratified atmosphere.

A particular case are ceilometers, characterized by emitting in the near infrared (usually between 900 and 1100 nm) using inexpensive pulsed laser diodes with a high pulse repetition frequency (PRF) and a pulse energy low enough to permit eye-safe operation [52]. Originally designed for cloud-base height determination, their use is rapidly growing due to their simplicity, small size, low cost and commercial availability. Several national weather services have set networks of ceilometers operating in quasi continuous-unattended regime and providing near real-time data. Although their capability to retrieve aerosol properties is at the moment constrained by technological limitations, this deployment has attracted the attention of the scientific community as complement of the existing networks of advanced lidar stations, with the aim of increasing both the spatial density of available aerosol data and the temporal continuity of observations [52,53].

Indeed, ceilometers suffer from significantly poorer signal-to-noise ratio than more advanced lidar systems. The detection of the molecular return at aerosol-free altitudes, required for the backscatter calibration at a reference height, becomes problematic and makes it difficult a correct quantitative retrieval of aerosol optical properties. Their current use is thus focused on the detection of cloud base heights and, regarding aerosol information, on vertical-structure profiling, which in turn allows to determine meteorological parameters like the mixing layer height using the aerosols as a proxy [54]. The basic functional scheme of a typical commercial ceilometer is the same as the one shown in Figure 1: it is formed by a pulsed laser diode as light source (typically 5–10 kHz PRF and 1–10 mJ pulse energy), an optical assembly to collect the backscattered radiation (100–200 mm diameter), a photodetector, commonly an avalanche photo diode (APD), and a digitizer board. Common performance parameters result in typical time and range resolutions of 5 min and 15 m and maximum range of 7.5 km.

### 3.2. 1β + 1α Vibro-Rotational and Pure Rotational Raman

**Figure 2:**
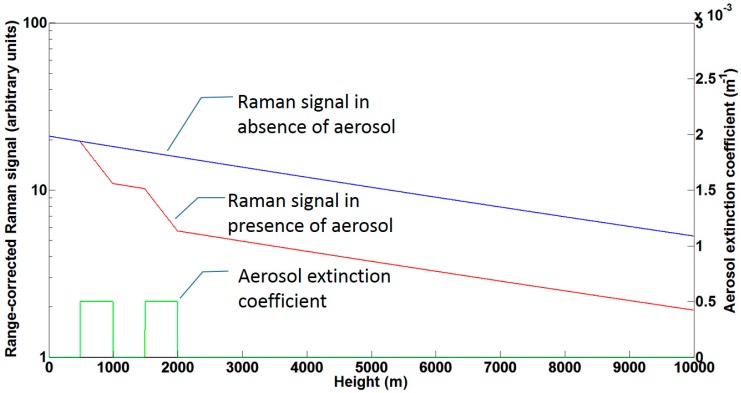
Illustration of the principle of the Raman technique to measure the aerosol extinction. The range-corrected Raman signal for a purely molecular atmosphere is represented by the blue curve. The red curve represents the range-corrected Raman signal if there were two aerosol layers (between 500 m and 1000 m, and between 1500 m and 2000 m) with $5\times 10^{-4}$ m^−1^ aerosol extinction coefficient (green curve).

In a purely elastic lidar the aerosol backscatter coefficient $\beta^{aer}\left(R\right)$ and the aerosol extinction coefficient $\alpha^{aer}\left(R\right)$ appear in an indistinguishable way—without more or less plausible further assumptions—in the expression of the received power in the term $\left[\beta^{aer}\left(R\right)+\beta^{mol}\left(R\right)\right]{\left[T^{aer}\left(R\right)T^{mol}\left(R\right)\right]}^{2}$(see Section 2 for the rest of the definitions). This is all the more unwelcome as the lidar ratio $S^{aer}\left(R\right)$ relating $\alpha^{aer}\left(R\right)$ and $\beta^{aer}\left(R\right)$ contains information about the physical composition and/or origin of the aerosol that one would like to measure, rather than to assume. To get over this issue and being able to determine independently the optical coefficients $\alpha^{aer}\left(R\right)$ and $\beta^{aer}\left(R\right)$, a number of solutions exist, one of the more widely extended [11,12] being the implementation of a channel measuring the backscattered radiation shifted by Raman effect from an abundant atmospheric species (N_2_ or O_2_) with well-defined proportion in the atmospheric composition. The principle lies in that, for a purely molecular atmosphere, the law followed by the molecule-specific Raman-shifted radiation collected by the lidar receiver is known, as it only depends (assuming it does not fall in the absorbing spectrum of an atmospheric gas) on the species number concentration and the molecular scattering; hence, departures from this known law can be related to the extinction introduced by the aerosols. This is illustrated in an idealized way in Figure 2. The blue curve represents the Raman return (for example from N_2_) if the atmosphere would not contain any aerosol, and depends solely on the number concentration of the species producing the Raman shift and the extinction originated by Rayleigh scattering. The effect of two assumed aerosol layers, one extending from 500 m to 1000 m, the other one from 1500 m to 2000 m, with aerosol extinction coefficient of $5\times 10^{-4}$ m^−1^ (green curve) is shown in the red curve. Note that the red curve is superimposed on the blue one before the first aerosol layer is encountered, and that the separation of both curves increases only when the aerosol extinction coefficient is non-zero, the rate of the separation increase (note the logarithmic scale) being proportional to the aerosol extinction coefficient. Assuming, as is the usual case in practice, that the Raman-shifting species is N_2_, the Raman lidar equation takes the form:

(5) $$P_{\lambda_{R}}\left(R\right)=\frac{K_{\lambda_{R}}}{R^{2}}\beta_{\lambda_{R}}\left(R\right)T_{\lambda_{0}}\left(R\right)T_{\lambda_{R}}\left(R\right)O_{\lambda_{R}}\left(R\right)$$

where $\beta_{\lambda_{R}}\left(R\right)$ is the N_2_ Raman backscatter coefficient (m^−1^·sr^−1^) at the Raman wavelength $\lambda_{R}$, βλR(R)=NN2(R)dσλR(π)dΩ with $N_{N_{2}}\left(R\right)$ the N_2_ molecule number density (m^−3^) and dσλR(π)dΩ the N_2_ Raman backscatter cross-section per solid angle unit (m^2^·sr^−1^). In contrast to Equation (3), the two-way path transmittance, $T_{\lambda_{0}}^{2}\left(R\right)$, is replaced by $T_{\lambda_{0}}\left(R\right)T_{\lambda_{R}}\left(R\right)$ where $T_{\lambda_{0}}\left(R\right)$ is the one-way atmospheric transmittance at $\lambda_{0}$ and $T_{\lambda_{R}}\left(R\right)$ is the atmospheric transmittance at $\lambda_{R}$ in the return path back to the lidar after Raman scattering has occurred.

Retrieval of the sought-after extinction-coefficient profile, $\alpha_{\lambda_{0}}^{aer}\left(R\right)$, can directly be tackled from the Raman channel alone (Equation (5)). In the presence of aerosols, the aerosol extinction emerges as a differential decrease in the two-way path atmospheric transmission, and can be expressed as:

(6) $$\alpha_{\lambda_{0}}^{aer}\left(R\right)=\frac{\frac{d}{dR}\ln\left\{\frac{N_{N_{2}}\left(R\right)\exp\left[-\int_{0}^{R}\left[\alpha_{\lambda_{0}}^{mol}\left(x\right)+\alpha_{\lambda_{R}}^{mol}\left(x\right)\right]dx\right]}{U_{\lambda_{R}}\left(R\right)}\right\}}{1+{\left(\frac{\lambda_{0}}{\lambda_{R}}\right)}^{\kappa }}$$

where κ=log(αλRaerαλRaer)/log(λ0λR) is the Ångström exponent expressing the $\lambda^{\kappa }$ spectral dependency of the aerosol extinction. The Ångström exponent is a property of the aerosol and is not known a priori, thereby introducing a source of uncertainty in the retrieved aerosol extinction coefficient. However, physically κ is in the range from −0.5 to 2 for most of aerosol types and $\lambda_{R}$ is close to $\lambda_{0}$, therefore the uncertainty introduced by κ through the term (λ0λR)κ in the denominator of Equation (6) is small.

Retrieval of the aerosol backscatter-coefficient profile, $\beta_{\lambda_{0}}^{aer}\left(R\right)$, is accomplished by forming the ratio Pλ0(R)PλR(Rm)Pλ0(Rm)PλR(R) and—as in the KF algorithm—by choosing a molecular reference height so that $\beta_{m}^{aer}\ll \beta_{m}^{mol}$, hence, $\beta_{m}\approx \beta_{m}^{mol}$. The aerosol backscatter-coefficient solution can be expressed in vector kernel form as:

(7) $${\vec{\beta }}_{\lambda_{0}}^{aer}=f\left[\underset{\begin{array}{l} channel\\ returns \end{array}}{\underset{︸}{{\vec{P}}_{\lambda_{0}},{\vec{P}}_{\lambda_{R}}}},{\vec{\alpha }}_{\lambda_{0}}^{aer},{\vec{N}}_{R},\underset{\begin{array}{l} Rayleigh\\ comp. \end{array}}{\underset{︸}{\vec{p},\vec{T}}},R_{m}\right]$$

where $\vec{p}$ and $\vec{T}$ are the pressure and temperature height-dependent profiles used to compute the molecular extinction coefficient and ${\vec{\alpha }}_{\lambda_{0}}^{aer}$ is the solution extinction profile of Equation (6), and *f* the function operator given by Equation (4) of [8].

**Figure 3:**
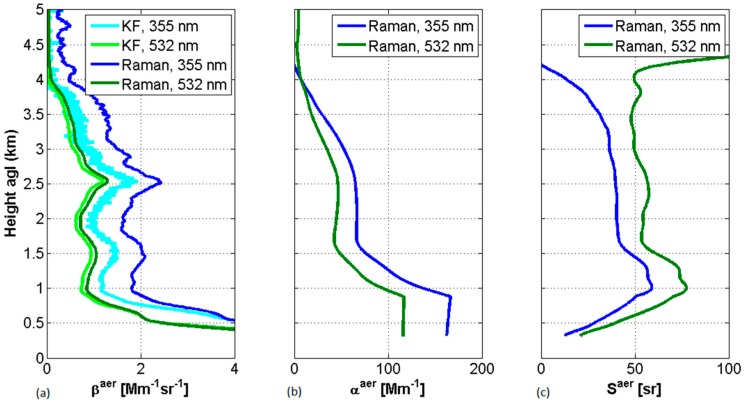
Example of retrievals of aerosol optical properties: (**a**) backscatter coefficient from KF (with a constant lidar ratio of 50 sr) and Raman algorithm; (**b**) extinction coefficient from Raman algorithm; and (**c**) lidar ratio from Raman algorithm. The data are from a Saharan dust intrusion detected in Barcelona, Spain, on 28 May 2016, with the multi-wavelength lidar system from the Universitat Politècnica de Catalunya (UPC).

In contrast to the KF retrieval method, the Raman lidar method is able to yield reliable extinction, backscatter and lidar-ratio profiles. Equation (6) is however hampered by the derivative nature of the Raman method, which leads to noisy retrievals of the extinction coefficient and which requires the use of noise-reduction techniques such as Savitzky–Golay and range-dependent spatial filtering, among others [55,56]. Another advantage of the method is its capability to retrieve the overlap function under the assumption that $O_{\lambda_{R}}\left(R\right)=O_{\lambda_{0}}\left(R\right)$ [57]. Figure 3 shows an example of retrieval of backscatter and extinction coefficients at 355 nm and 532 nm with the Raman algorithm, compared with a KF inversion using a constant lidar ratio of 50 sr. The lidar ratio, the extinction-to-backscatter ratio, a product derived from the Raman inversion, is also plotted on panel (c). One sees that above 1.5 km both lidar ratios at 355 nm and 532 nm are oscillating around values close to 50 sr, a rather typical value for mineral dust [58]. The retrieval of the backscatter coefficients with the Raman algorithm look smoother because spatial filtering was applied.

Traditionally, lines of the N_2_ vibro-rotational Raman spectrum have been used to implement this technique, because the shifted wavelengths are well separated from the emitted one (e.g., under the excitation of a 355 nm emitted wavelength from a frequency-tripled Nd: YAG laser, the Raman-shifted wavelength is 387 nm), allowing a relatively easy separation in the receiver of the Raman-shifted return from the elastic one at the emitted wavelength using optical interference filters. However this method has the drawback of the extremely low values of the differential cross-section of lines in the vibrational Raman spectrum. This results in noisy returns, far from the smooth idealized ones on Figure 2, impairing the inversion and actually making it impossible, for most of the systems working according to this principle, to operate in daytime conditions, because of the noise added to the received signal by the sky background radiation passing through the optical filter.

To overcome this problem, the pure rotational Raman spectrum of N_2_ and O_2_, presenting higher (around two orders of magnitude) differential cross-section than the vibrational one, can be used. This adds the advantage of suppressing the effect of the uncertainty on the Ångström exponent, as the lines of the pure rotational Raman spectrum are very close to the excitation wavelength, but, for this same reason, the price has to be paid of higher technical difficulty, as very selective filtering devices are required to reject the elastic return, which is contaminated by that of aerosols; moreover the dependence on temperature of the backscatter differential cross section of the lines of the pure rotational Raman spectrum must be taken into account. However, technological progress is making available interference filters that can be tailored to select portions of the N_2_ an O_2_ pure rotational Raman spectrum with low temperature dependence of the scattering cross section [59], while providing sufficient rejection of the elastic backscatter wavelength.

### 3.3. HSRL

**Figure 4:**
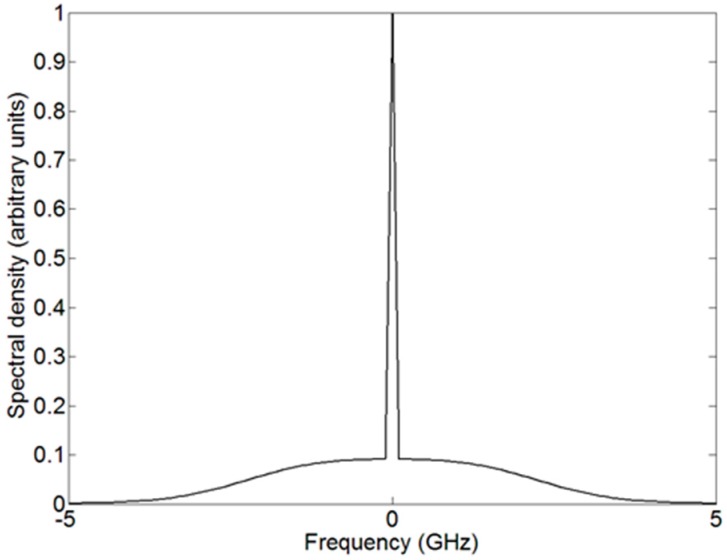
Qualitative backscatter spectrum around the central backscatter frequency of an atmosphere containing aerosol. The frequencies are referred to the one emitted by the laser.

In the quest of ways for increasing the reference signal to determine the aerosol extinction coefficient, high-spectral-resolution lidars (HSRL, [60,61,62,63]) use the elastic Rayleigh scattering itself, with cross sections more than one order of magnitude higher than those of the pure rotational Raman spectrum, as reference signal (equivalent to the blue curve in Figure 2). To exploit this, the elastic return from molecules (Rayleigh scattering) must be separated from that from aerosols. This is done building on the fact that molecules are subject to thermal motion, their elastic backscattering spectrum being therefore widened by Doppler effect, while the backscatter from aerosols is much narrower spectrally. This is qualitatively illustrated in Figure 4, showing the aspect of the elastic return spectrum around the emitted wavelength. The central peak with virtually no frequency widening corresponds to the aerosol return, while the wings, extending several GHz above and below, are the return from the molecules with a Maxwell’s distribution of velocities (although other effects, such as Brillouin scattering [64], can also affect the shape of the widened molecular contribution to the return spectrum). 

**Figure 5:**
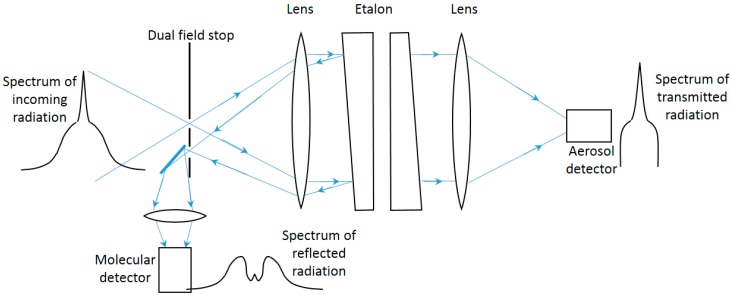
Principle of the HSRL using an etalon to separate the molecular backscatter from the aerosol one (adapted from [62]).

The separation needs a very selective optical filter, usually implemented with a Fabry-Pérot etalon passing the non-widened return from the aerosol and reflecting most of the widened molecular backscatter (Figure 5, [62]), or with a molecular absorption cell absorbing the aerosol backscatter. While more sensitive than the Raman one, this technique requires more complexity in the system, as the lasers must be injection seeded to achieve the necessary spectral purity and their frequency must be locked to that of the Fabry-Pérot etalon or to that of the molecular filter.

### 3.4. Depolarization-Sensitive System

**Figure 6:**
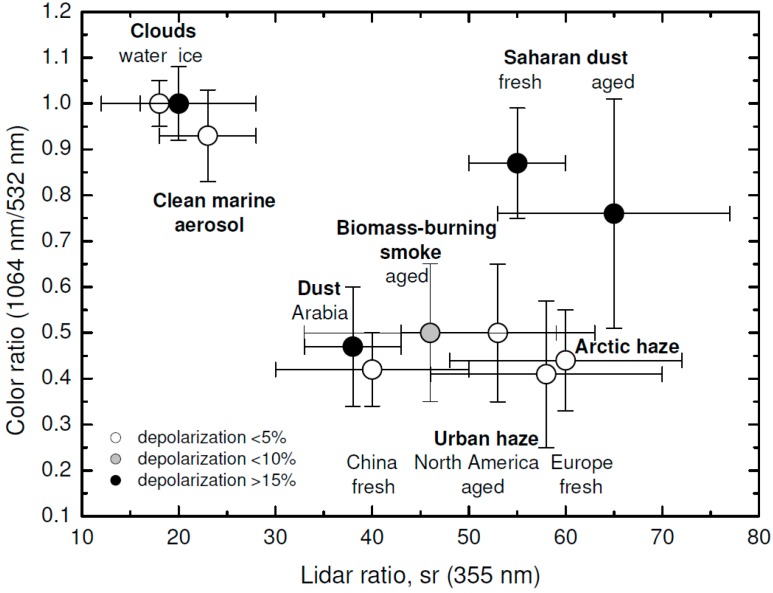
Color ratio vs. lidar ratio vs. depolarization ratio for different aerosol and cloud types [72] with data from [73].

The lidar depolarization technique [65,66,67] is a mature method that permits identifying different aerosol types present in the atmosphere: ice crystals in clouds [67], contrails [68,69], Saharan dust [70] or hydrometeors [71]. The main objective of this technique is the retrieval of the particle linear depolarization ratio, $\delta^{p}$, defined as [70]:

(8) $$\delta^{p}=\frac{\beta_{⊥}^{aer}}{\beta_{\left|\right|}^{aer}}$$

where $\beta_{⊥}^{aer}$ and $\beta_{∥}^{aer}$ stand for the aerosol cross-polar and co-polar backscatter coefficients, respectively. The particle depolarization ratio, together with the lidar ratio (see Section 3.1) and the color ratio (relation between backscatter coefficients at different wavelengths) give a good approach to identify the type of atmospheric aerosol [72]. Figure 6 shows the different values for the three indicated parameters for some of the most common aerosol types found in the atmosphere.

The particle depolarization ratio cannot be measured directly because what the lidar systems measure is the light backscattered by both the particles and molecules. So, the primary measured magnitude is the volume depolarization ratio, $\delta^{V}$, defined as [70]:

(9) $$\delta^{V}=\frac{\beta_{⊥}}{\beta_{∥}}$$

where $\beta_{⊥}$ and $\beta_{∥}$ are, respectively, the total cross-polar and co-polar backscatter coefficients, i.e., those measured when the received light is passed through polarizers oriented perpendicular or parallel to the polarization of the emitted beam.

If the backscatter ratio $BR=\left(\beta^{aer}+\beta^{mol}\right)/\beta^{mol}$ has been calculated from an elastic or Raman inversion, the particle depolarization ratio can be calculated as [70]:

(10) $$\delta^{p}=\frac{\left(1+\delta^{m}\right)\delta^{V}BR-\left(1+\delta^{V}\right)\delta^{m}}{\left(1+\delta^{m}\right)BR-\left(1+\delta^{V}\right)}$$

where $\delta^{m}$ is the linear depolarization ratio of air molecules, which can be determined previously as a function of temperature and optical bandwidth of the receiver [74]. Regarding the volume depolarization ratio retrieving instrumentation, two main approaches can be found in the lidar community: in the first one the lidars measure the cross- and co-polar signals [25]; in the other one the lidars measure the total power and cross-polar signals [75,76]. In all cases, when total or co-polar and cross-polar signals are detected in two different channels, the calculation of the volume depolarization ratio requires a system calibration between both channels [70].

**Figure 7:**
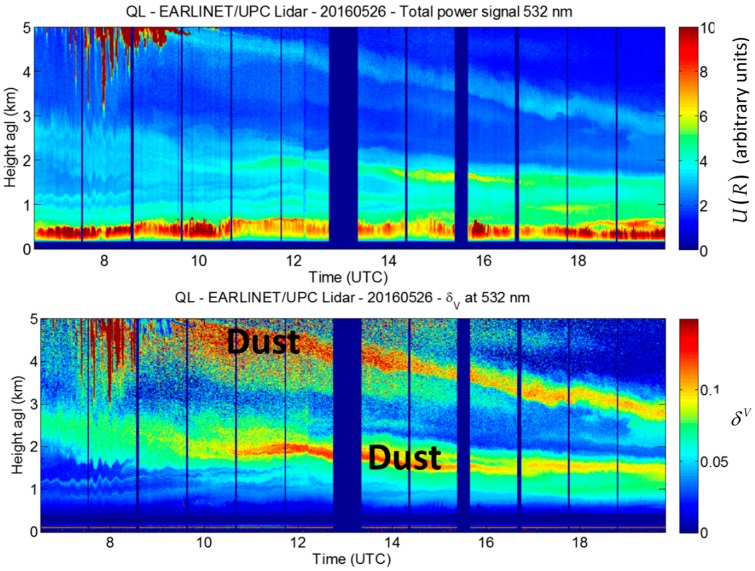
Detection of Saharan dust with depolarization channel with the UPC multi-wavelength lidar during the 26 May 2016 intrusion: (**a**) Range-corrected total power as a function of time and height above ground level; (**b**) Volume depolarization ratio as a function of time and height.

**Figure 8:**
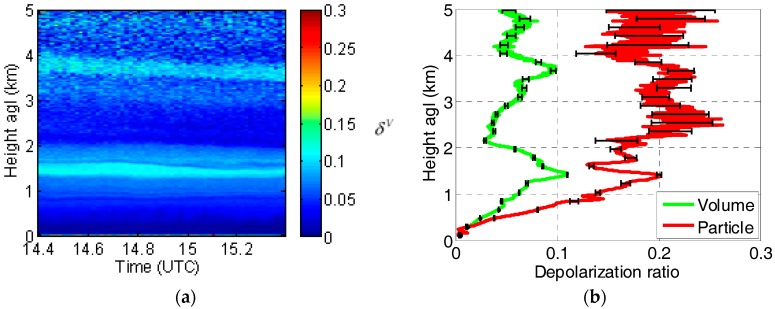
Depolarization ratios measured with the UPC multi-wavelength lidar during the 26 May 2016 Saharan dust intrusion event: (**a**) quicklook of the volume depolarization ratio between 14:24 and 15:23 UT with a time resolution of 1 min.; (**b**) average vertical profile of the volume (green) and particle (red) depolarization ratios with their associated error bars (black).

As an example, Figure 7 shows a quick look plot of the range-corrected total power and of volume depolarization ratio, both vs. time and height, measured by the UPC multi-wavelength lidar system during a strong Saharan mineral dust event over Barcelona, Spain, on 26 May 2016. While the range-corrected total power $(U\left(R\right)=R^{2}P\left(R\right)$ upper panel) is only able to reveal the multi-layering aspect of the low troposphere, the high values (yellow to orange/red color) of the volume depolarization ratio ($\delta^{V}$, lower panel) puts in evidence the regions where dust is present. From the same event, Figure 8 shows the profiles of volume and particle depolarization ratio averaged over 1 h, starting at 14:24 UTC. Dust is present at all heights above 0.5 km. The particle depolarization ratio is quite variable since it ranges between 15% and 25% above 1 km.

### 3.5. Multi-Wavelength Lidars

Multi-wavelength lidars are systems recording the backscattered signal at several wavelengths, which can be combined together to form advanced aβ + bα + cδ systems capable of measuring a backscatter coefficients at different wavelengths, b extinction coefficients and c particle depolarization ratios. Two types of advanced systems are commonly used:

- Multi-wavelength elastic, vibro-rotational Raman and polarization-sensitive systems
- Multi-wavelength HSRL and polarization-sensitive systems

Although multi-wavelength pure rotational Raman systems are in principle feasible, they are left apart in this list because the technique is rather new and no such system exists to our knowledge at the time of writing of this article. The system reported in [59] uses two variants of the Raman technique but at a single elastic wavelength: elastic/vibro-rotational Raman and elastic/pure rotational Raman, to evaluate the advantages brought by recent elastic/pure rotational Raman systems vs. the classical elastic/vibro-rotational Raman systems (see Section 3.2). The Backscatter Extinction Ratio Temperature, Humidity Lidar (BERTHA) lidar from the Leibniz Institute for Tropospheric Research (TROPOS, Leipzig, Germany) is configured in [77] into a two elastic/vibro-rotational Raman + one elastic/pure rotational Raman, to evaluate the retrieval of the extinction coefficient at 1064 nm. The LIlle Lidar AtmosphereS (LILAS) system [78] from the Laboratoire d’Optique Atmospherique (LOA, Villeneuve d’Ascq, France) is a 3β + 2α + 3δ system and is an example of a rather complete multi-wavelength lidar system.

The ability of multi-wavelength systems of providing information on the spectral dependency of the aerosol optical properties, in combination with depolarization data, makes them suitable to further retrieve information on the aerosol microphysics. This ability requires a minimum configuration of 2β + 2α, being the configuration of 3β + 2α + 1δ a good trade-off between information retrieved and system complexity, allowing to retrieve range-resolved particle effective radius, radius distribution, volume concentration, and refractive index [16,19].

## 4. Conclusions

Lidars are powerful sensors for range-resolved atmospheric sounding. In particular, aerosol lidars have reached a considerable degree of maturity, which allows, through coordinated ground-based lidar networks and spaceborne orbiting lidars, to gain, on the one hand, insight in the role of aerosols and clouds in the global radiative balance, and, on the other hand, to contribute to air-quality forecasts and meteorological analyses.

## Figures and Tables

**Table 1:** Aerosol properties that can be retrieved from aerosol lidar measurements. An aβ + bα + cδ system is formed by a elastic channels, b Raman channels and c depolarization channels. r_eff_ is the effective radius of the aerosol size distribution, ω_0_ the single scattering albedo, C the mass concentration, V the volume concentration and n the refractive index. The suffix m indicates the size distribution mode (fine or coarse) and the suffix s indicates the sphericity of the particles (spherical or spheroid). * MODIS: Moderate Resolution Imaging Spectroradiometer on board NASA satellites.

Properties	Parameters		Configuration	References
Structural	PBL height		1β and >	[3,4]
	Lofted layer base, top and thickness		1β and >	
	Cloud base, top and thickness		1β and >	[5]
Optical	Backscatter coefficient		1β and >; multi-angular	[6,7,8,9,10]
	Extinction coefficient		1β + 1α and >	[11,12]
Microphysical	Shape		1β + 1δ	[13]
	Size	r_eff_, ω_0_, C, n domain	3β + 2α	[14,15,16,17,18,19]
		β_m,s_, α_m,s_, C_m,s_, V_m,s_	3β + 1δ + Sun-Photometer	[20,21,22]
		r_eff_	2β + MODIS *-derived optical properties	[23]
		β_m_, C_m_	1β + 1δ if 2 or 3 aerosol types of different depolarization ratios can be identified	[24,25,26]
